# Basal DNA repair machinery is subject to positive selection in ionizing-radiation-resistant bacteria

**DOI:** 10.1186/1471-2164-9-297

**Published:** 2008-06-21

**Authors:** Haïtham Sghaier, Kaïs Ghedira, Alia Benkahla, Insaf Barkallah

**Affiliations:** 1 Unit of Microbiology and Molecular Biology, National Center for Nuclear Sciences and Technologies (CNSTN), Sidi Thabet Technopark, 2020 Sidi Thabet, Tunisia; 2 Group of Bioinformatics and Modelling, Laboratory of Immunology, Vaccinology, and Molecular Genetics, Institute Pasteur of Tunis, 13, place Pasteur BP 74, 1002 Tunis, Tunisia

## Abstract

**Background:**

Ionizing-radiation-resistant bacteria (IRRB) show a surprising capacity for adaptation to ionizing radiation and desiccation. Positive Darwinian selection is expected to play an important role in this trait, but no data are currently available regarding the role of positive adaptive selection in resistance to ionizing-radiation and tolerance of desiccation. We analyzed the four known genome sequences of IRRB (*Deinococcus geothermalis*, *Deinococcus radiodurans*, *Kineococcus radiotolerans*, and *Rubrobacter xylanophilus*) to determine the role of positive Darwinian selection in the evolution of resistance to ionizing radiation and tolerance of desiccation.

**Results:**

We used the programs MultiParanoid and DnaSP to deduce the sets of orthologs that potentially evolved due to positive Darwinian selection in IRRB. We find that positive selection targets 689 ortholog sets of IRRB. Among these, 58 ortholog sets are absent in ionizing-radiation-sensitive bacteria (IRSB: *Escherichia coli *and *Thermus thermophilus*). The most striking finding is that all basal DNA repair genes in IRRB, unlike many of their orthologs in IRSB, are subject to positive selection.

**Conclusion:**

Our results provide the first *in silico *prediction of positively selected genes with potential roles in the molecular basis of resistance to γ-radiation and tolerance of desiccation in IRRB. Identification of these genes provides a basis for future experimental work aimed at understanding the metabolic networks in which they participate.

## Background

In this paper, we consider ionizing-radiation-resistant bacteria (IRRB) as "non-spore-forming bacteria that can protect their cytosolic proteins from oxidation and tolerate many DNA double-strand breaks (DSBs) after exposure to high, acute ionizing radiation (dose greater than 1 kilogray (kGy) for 90% reduction (D_10_) in Colony Forming Units (CFUs)), and can resist prolonged desiccation."

Over the past five decades, *Deinococcus radiodurans *(D_10 _≈ 15 kGy) has been a model for understanding many of the basic principles that govern resistance to ionizing radiation and tolerance of desiccation (for review see [1]). Recent findings indicated that the mutual nature of *D. radiodurans*'s γ-radiation resistance and desiccation tolerance resides in cytosolic Mn-dependent mechanisms that protect proteins from oxidative modifications that introduce carbonyl groups [2,3]. Like most IRRB, *D. radiodurans *accumulates about 300 times more Mn(II) than ionizing-radiation-sensitive bacteria (IRSB) [4] such as *Escherichia coli *(D_10 _≈ 0.7 kGy) and *Thermus thermophilus *(D_10 _≈ 0.8 kGy) [5,6]. Due to its high concentration of intracellular Mn(II) ions and the consequent protection of proteins, *D. radiodurans *can survive 10 kGy of ionizing radiation, a dose that causes approximately 100 DNA DSBs per genome. This species can also survive 8 days in a desiccator with no obvious DNA DSBs [2-4,7,8]. Early on, analysis of *D. radiodurans*'s transcriptional response revealed that the cellular responses to ionizing radiation and desiccation exhibit substantial overlap [9]. In this context, 41 ionizing-radiation-sensitive strains of *D. radiodurans *were also shown to be sensitive to desiccation [7]. Furthermore, the mutational inactivation of the genes DR_1172 – encodes a homolog of LEA76, a group 3 LEA (late embryogenesis abundant) protein – and DR_B0118 – encodes a protein that is expressed during dehydration by the resurrection plant, *Craterostigma plantagineum *– greatly sensitized *D. radiodurans *to desiccation, but not to ionizing radiation [10]. The former result based on a collection of ionizing-radiation-sensitive strains of *D. radiodurans *is consistent with evidence that dried bacterial cells exhibit a substantial number of DNA DSBs, single-strand breaks, and DNA crosslinks [11], DNA damage that is also observed following exposure to ionizing radiation [12]. Indeed, ionizing radiation and desiccation introduce similar types of DNA damage – DNA DSBs – in *D. radiodurans *[7]. The latter result based on the mutational inactivation of the genes DR_1172 and DR_B0118 considerably strengthens the hypothesis that the *D. radiodurans*'s desiccation tolerance could be a consequence of this organism's adaptation to ionizing radiation (radiation adaptation hypothesis), particularly that the origin of ionizing-radiation resistance in bacteria can be explained as an adaptation to environmental radiation [13]. For example, *Deinococcus *sp. have been isolated from the subsurfaces of hydrothermal vents at depths of 64.8–128.9 m below the sea floor (mbsf) [14]. D'Hondt and colleagues [15] have recently surveyed environments representative of a broad range of subsurface conditions that can be found in marine sediments. Among the most striking features of deeply buried sediments (20–100 mbsf) are Mn-rich sites with high natural γ-radiation levels. Ionizing-radiation levels in such deposits might have been much higher on ancient Earth (Daly MJ, personal communication, 2006). Concerning the close relationship between bacterial ionizing-radiation resistance and desiccation tolerance [2,3,7,9], many of the environments from which IRRB have been isolated can be considered to be dry, and it has been shown that many of these strains are also desiccation tolerant [16]. Moreover, it was demonstrated that IRRB are present at higher numbers in an arid soil than in a nonarid soil, and that IRRB in the arid soil are recovered after exposure to higher doses than the doses which allow recovery of bacteria from the nonarid soil [16]. In this context, many IRRB (i.e. *Deinococcus deserti*, *Deinococcus sonorensis*, *etc*.) have been isolated from the desert [16,17], indicating they have the capacity to adapt to harsh environments.

Three principal mechanisms may have contributed to the adaptability of IRRB: positive Darwinian selection, lateral gene transfer, and gene regulation [18]. Previous studies have discussed the importance of lateral gene transfer and gene regulation in *Deinococcus *evolution [5,6]. However, no previous research has addressed the role of positive Darwinian selection in resistance to ionizing-radiation and tolerance of desiccation in IRRB. In this paper, we assess the role of positive Darwinian selection by analysis of the genomes of IRRB. Among the many IRRB, completely sequenced genomes are only available for *D. geothermalis *(D_10 _≈ 15 kGy), *D. radiodurans*, *Kineococcus radiotolerans *(D_10 _≈ 2 kGy), and *Rubrobacter xylanophilus *(D_10 _≈ 5.5 kGy) [1,6,19,20]. When bacterial species of the genera *Deinococcus *(*D. geothermalis *and *D. radiodurans*) and *Kineococcus *(*K. radiotolerans*) were tested *in vitro *for desiccation tolerance, all were found to be tolerant to desiccation [4,21]. We are not aware of any studies that examined *R. xylanophilus *for desiccation tolerance, but since (1) many IRRB isolated from the desert – they are one way or another tolerant to desiccation – cluster within the monophyletic *Rubrobacteria *subgroup which is specialized for particular niches present in arid soils [22,23], (2) bacterial species of the genus *Rubrobacter *(*R. radiotolerans*, *R. taiwanensis*, and *R. xylanophilus*) belong to IRRB [24,25], and (3) there has to our knowledge never been a report of desiccation-sensitive IRRB, we included *R. xylanophilus *in our study.

Positive Darwinian selection leads to the fixation of advantageous mutations and is the fundamental process behind adaptive changes in genes (reference [26] and citations therein). Many statistical methods have been developed for detecting adaptive molecular evolution [27-29], such as the "conservative" PAML program [30,31]. In this report, we used a freely available software package with a user-computer interface (DnaSP) [32] to identify genes under positive selection in IRRB. This program calculates the ratios of nonsynonymous to synonymous mutation rates (Ka/Ks) in protein coding genes. The Ka/Ks ratio measures the strength of selection, with values > 1 indicative of positive selection [29].

## Results and discussion

At the time we began this work, the GOLD database [20] documented four completely sequenced IRRB genomes: *D. geothermalis *DSM 11300 [6], *D. radiodurans *R_1 _[19], *K. radiotolerans *SRS30216 [21], and *R. xylanophilus *DSM 9941 [24]. We identified 734 orthologs that were present in all four species. To validate our subsequent results, we deduced hits in IRSB genomes (*Escherichia coli *536, *Escherichia coli *K-12, *Thermus thermophilus *HB8, and *Thermus thermophilus *HB27) of the 734 ortholog sets in IRRB [see Additional file 1]. Then, we used DnaSP to examine the aligned ortholog sets among IRRB [see Additional files 2, 3, 4, 5, 6, 7, 8] and IRSB [see Additional files 9, 10, 11] to search for evidence of positive selection [32]. We identified 689 ortholog sets among IRRB as potentially subject to positive Darwinian selection (Additional file 1, genes in bold). Supporting our conclusion that these genes have been under selective pressure from niches with high natural γ-radiation or desiccation levels [13], we found that many of these positively selected genes are involved in resistance to γ-radiation resistance and tolerance of desiccation [6,9]. To find out if there were any biais due to phylogenetic relationships, patterns of positive selection in *Deinococcus *and *Thermus *species were compared, because phylogenetically the closest relative of *Deinococcus *is the genus *Thermus *[5]. As patterns of positive selection in *Deinococcus *and *Thermus *species were different [see Additional file 1], we concluded that DnaSP scores are related to resistance to ionizing-radiation and tolerance of desiccation phenotypes and not to the phylogenetic relationships of the species. But it should be noted that our bioinformatical method used to detect positive selection is based on sequence alignments, and therefore we cannot exclude that in some cases we have included false positives because of improper alignments.

We defined two important families of positively selected genes in IRRB: i) Group-I genes, which are under positive selection in IRRB but under neutral or purifying selection in IRSB (125 genes, highlighted in light gray in Additional file 1), and ii) Group-II genes, which are under positive selection in IRRB but absent in all IRSB (58 genes, highlighted in dark gray in Additional file 1). These two groups of positively selected genes in IRRB may provide insight into the molecular adaptations to ionizing radiation and desiccation. For instance, Group-II includes DNA repair-related proteins (DR_0192, MutT/NUDIX family protein; DR_2074, putative 3-methyladenine DNA glycosylase), oxidative damage-related proteins (DR_2242, thiol-specific antioxidant protein; DR_2538, cytochrome P450) and water stress-associated proteins (DR_0463, maltooligosyltrehalose synthase; DR_0464, putative maltooligosyltrehalose trehalohydrolase). These conclusions are in concordance with DNA-centric hypotheses (for example, [33]), protein-centric hypotheses [2,3], and the water replacement with trehalose hypothesis [34] as protection mechanisms against ionizing radiation and desiccation. Our results support the idea that resistance to ionizing radiation and tolerance of desiccation are two complex phenotypes, and suggest that protection and repair mechanisms are complementary in IRRB.

The *D. radiodurans *genome has most of the DNA repair proteins found in *E. coli *[19]. Broad-based bioinformatic and experimental studies have concluded that *D. radiodurans *uses a relatively conventional set of DNA repair and protection mechanisms, but that these mechanisms are much more efficient than in IRSB [2,3,9,19]. We found that some of the accessory DNA repair genes in *Deinococcus *that are extremely important for resistance phenotypes (e.g., *pprI *(DR_0167, Dgeo_0395) and *pprA *(DR_A0346, Dgeo_2628) [35,36]) have no orthologs in *Kineococcus *and *Rubrobacter*. Similarly, the five transcripts of *D. radiodurans *(*ddrA*, DR_0423; *ddrB*, DR_0070; *ddrC*, DR_0003; *ddrD*, DR_0326; *pprA*, DR_A0346) that are most highly induced following ionizing radiation and recovery from desiccation [9] were not present in *Kineococcus *and *Rubrobacter*.

It seems likely that the shared ability of IRRB to survive the damaging effects of ionizing radiation and desiccation is the result of basal DNA repair pathways and that basal DNA repair genes have been acted upon by positive selection. Table 1 shows that, unlike many of their orthologs in IRSB [37], all DNA replication, repair, and recombination genes in IRRB were subject to positive selection. Three major DNA repair genes showed remarkably strong evidence for positive selection: DR_1707 (Ka/Ks = 3.15), DR_0906 (Ka/Ks = 3.4) and DR_1913 (Ka/Ks = 3.48).

**Table 1:** Replication, repair, and recombination genes under positive selection in ionizing-radiation-resistant bacteria (IRRB)^a^.

**Orthologs**	**Function**	**(Ka/Ks)**^b^	**D value (Tajima test)**^b^
	**DNA polymerase**		

DR_0467 Dgeo_1609 Krad_R0056 Rxyl_0486	Hypothetical DNA polymerase^♠^	n.a.^c^	-1.779
DR_0507 Dgeo_0255 Krad_3187 Rxyl_1096	DNA polymerase III, α subunit*	n.a.	-1.725
DR_0856 Dgeo_1818 Krad_3247 Rxyl_2984	DNA polymerase III, ε subunit◆	2.81	
DR_1244 Dgeo_0745 Krad_3423 Rxyl_1518	Putative DNA polymerase III, δ subunit◆	1.68	
DR_1707 Dgeo_1666 Krad_2951 Rxyl_2025	DNA-directed DNA polymerase*	3.15	
DR_1751 Dgeo_1556 Krad_1521 Rxyl_0503	DNA polymerase-related protein◆	n.a.	-1.744
DR_2410 Dgeo_2135 Krad_R0007 Rxyl_2444	DNA polymerase III, τ/γ subunit*	2.3	

	**Replication complex**		

DR_0100 Dgeo_0165 Krad_4338 Rxyl_0045	Single-stranded DNA-binding protein*	n.a.	-1.872
DR_0549 Dgeo_2037 Krad_4333 Rxyl_0852	Replicative DNA helicase^♠^	n.a.	-1.539
DR_0601 Dgeo_R0043 Krad_3361 Rxyl_1502	DNA primase*	n.a.	-1.770
DR_0906 Dgeo_0546 Krad_0006 Rxyl_0005	DNA gyrase, subunit B*	3.4	
DR_1374 Dgeo_2001 Krad_0487 Rxyl_1964	DNA topoisomerase I*	3.13	
DR_1913 Dgeo_1016 Krad_0007 Rxyl_0006	DNA gyrase, subunit A*	3.48	

	**Other DNA-associated proteins**		

DR_0120 Dgeo_2345 Krad_1409 Rxyl_1396	smf protein^♠^	1.87	
DR_0289 Dgeo_0248 Krad_0422 Rxyl_2676	Endonuclease III^♠^	2.82	
DR_0440 Dgeo_0327 Krad_3056 Rxyl_1322	Holliday junction resolvase*	2.06	
DR_0493 Dgeo_0442 Krad_1377 Rxyl_2433	Formamidopyrimidine-DNA glycosylase◆	2.37	
DR_0596 Dgeo_0404 Krad_3053 Rxyl_1324	Holliday junction DNA helicase*	n.a.	-1.789
DR_1089 Dgeo_1620 Krad_0004 Rxyl_0004	RecF protein*	n.a.	-1.342
DR_1105 Dgeo_1212 Krad_0603 Rxyl_2178	DNA repair protein*	2.61	
DR_1274 Dgeo_0726 Krad_3054 Rxyl_1323	Holliday junction binding protein^♠^	1.88	
DR_1354 Dgeo_1124 Krad_2935 Rxyl_2010	Excinuclease ABC, subunit C*	n.a.	-1.411
DR_1477 Dgeo_1194 Krad_3147 Rxyl_1453	DNA repair protein^♠^	2.08	
DR_1532 Dgeo_0545 Krad_1067 Rxyl_0909	Transcription-repair coupling factor*	3.05	
DR_1771 Dgeo_0694 Krad_2940 Rxyl_2016	Excinuclease ABC, subunit A^♠^	n.a.	-1.678
DR_1775 Dgeo_0868 Krad_0757 Rxyl_0825	DNA helicase II*	n.a.	-1.450
DR_1916 Dgeo_1139 Krad_1368 Rxyl_1371	DNA helicase RecG*	3.35	
DR_1921 Dgeo_0824 Krad_2554 Rxyl_0449	Exonuclease SbcD, putative◆	2.36	
DR_1922 Dgeo_0823 Krad_2553 Rxyl_0448	Exonuclease SbcC^♠^	1.82	
DR_1949 Dgeo_1623 Krad_1405 Rxyl_1394	Ribonuclease HII^♠^	n.a.	-1.642
DR_2074 Dgeo_1660 Krad_3154 Rxyl_1309	Putative 3-methyladenine DNA glycosylase♣	3.25	
DR_2275 Dgeo_1890 Krad_2942 Rxyl_2021	Excinuclease ABC, subunit B*	n.a.	-1.767
DR_2285 Dgeo_0019 Krad_0599 Rxyl_2229	A/G-specific adenine glycosylase*	1.85	
DR_2340 Dgeo_2138 Krad_1492 Rxyl_1423	RecA protein*	n.a.	-1.179
DR_2584 Dgeo_0107 Krad_4325 Rxyl_1215	DNA-3-methyladenine glycosidase II, putative*	n.a.	-1.607

^a^IRRB are *Deinococcus geothermalis*, *Deinococcus radiodurans*, *Kineococcus radiotolerans *and *Rubrobacter xylanophilus*.

^b^Positive selection: (Ka/Ks) > 1 or Tajima's D < 0.

^c^not available. See Methods.

^♠^Genes under positive selection in IRRB and under neutral or purifying selection in ionizing-radiation-sensitive bacteria (IRSB). *Genes under positive selection in both IRRB and IRSB.

◆ Genes under positive selection in IRRB for which orthologs were present in some, but not all, IRSB.

♣ Genes under positive selection in IRRB for which there are no orthologs in all IRSB.

See Methods and Additional file 1.

Zahradka et al. [33] reported the remarkable efficiency of DNA repair enzymes in *D. radiodurans *during recovery from ionizing radiation and proposed a model named 'Extended Synthesis-Dependent Strand Annealing' (ESDSA). This model proposes that DNA polymerase I (PolA, DR_1707) accounts for the high fidelity of RecA-dependent DNA DSB fragment assembly. *recA *(DR_2340), a central gene to genomic restoration, is up-regulated in *D. radiodurans *cultures recovering from ionizing radiation and desiccation [9], and Table 1 shows that *recA *is positively selected. In particular, PolA of *D. radiodurans *supports very efficient DNA replication at the earliest stages of recovery and is present at higher levels than during normal DNA replication [33]. It is unclear whether *polA *paralogs participate in ESDSA. However, the presence of *polA *paralogs that are subject to positive selection (unlike orthologs in IRSB) suggests that these paralogs may be involved in genomic networks of resistance to γ-radiation and tolerance of desiccation (see Table 1). A positively selected *polA *paralog set might also explain the results of Gutman et al. [38], who showed that ionizing-radiation-sensitive *D. radiodurans pol*A mutants are fully complemented by expression of the *pol*A gene from the relatively ionizing-radiation sensitive *E. coli*. A more complete examination of these paralogous proteins will be necessary to prove this hypothesis.

Genes encoding the two subunits of the DNA gyrase, DR_1913 (*gyrA*) and DR_0906 (*gyrB*), are also induced by ionizing radiation and desiccation [9], and this presumably explains their strong positive selection values. This implies that DNA supercoiling is important for DNA repair following the deleterious effects of ionizing radiation and desiccation.

Four additional proteins involved in DNA repair are also subject to strong positive selection: (1) DR_1374 (Ka/Ks = 3.13; DNA topoisomerase I, Uniprot Q9RUL0), (2) DR_1532 (Ka/Ks = 3.05; a transcription-repair coupling factor, Uniprot Q9RU62), (3) DR_1916 (Ka/Ks = 3.35; RecG helicase likely to be involved in DNA DSB repair, Uniprot Q9RT50), and (4) DR_2074 (Ka/Ks = 3.25; putative 3-methyladenine DNA glycosylase, Uniprot Q9RSQ0). This is likely the result of continuous interaction between IRRB and natural niches, which have high levels of γ-radiation or high probability of desiccation [13-17].

The present work has identified the first suite of genes that are under positive Darwinian selection in IRRB. Our results can serve as a useful background to guide future physiological and biochemical experiments examining resistance to ionizing radiation and tolerance of desiccation. As more IRRB genome sequences become available, particularly in more distantly related species, we expect that our methods will provide a sensitive and controlled approach for detection of genes that have been subject to positive Darwinian selection for resistance to ionizing radiation and tolerance of desiccation.

## Methods

To identify genes under positive selection in IRRB, the DnaSP program requires an aligned set of orthologous sequences [32]. Thus, we initiated our analyses by performing comparisons between all fully sequenced IRRB genomes (*Deinococcus geothermalis *DSM 11300 [6], *Deinococcus radiodurans *R_1 _[19], *Kineococcus radiotolerans *SRS30216 [21], and *Rubrobacter xylanophilus *DSM 9941 [24]). Information on complete and ongoing IRRB genome sequencing projects was obtained from the GOLD database [20]. Genome sequences were downloaded from the National Center for Biotechnology Information (NCBI) RefSeq repository [39]. For comparison, we used the genomes of 4 IRSB: *Escherichia coli *536 (UPEC), *Escherichia coli *K-12 MG1655, *Thermus thermophilus *HB8, and *Thermus thermophilus *HB27 [20,39]. MultiParanoid was used to find orthologous relationships [40]. Orthologous sequences were imported into Bioedit Sequence Alignment Editor (version 7.0.9) [41], and the sequences for each ortholog set were aligned with CLUSTAL W [42]. To test for positive selection, we used DnaSP (version 4.0) [32]. Using the Jukes and Cantor method [43], DnaSP calculates the nonsynonymous (Ka) and synonymous (Ks) substitution rates for each codon. When the nonsynonymous rate was greater than the synonymous rate (Ka/Ks > 1), this is indicative of positive Darwinian selection [29,32]. When it was not possible to calculate Ka or Ks, we calculated Tajima's D value (in addition to a statistical significance P value), with the confidence limit of D equal to 99.9%. Negative values of Tajima's D indicated positive selection [27,44].

## Authors' contributions

HS, KG, AB and IB designed the study. HS and KG implemented the methods, analyzed the data, and wrote the manuscript. HS and IB analyzed all biological data. KG and AB supervised and oversaw all computational aspects. All authors have read and approved the final version of the manuscript.

## Supplementary Material

Additional file 1 **Common genes in ionizing-radiation-resistant bacteria (IRRB) and positive Darwinian selection**. Bold letters denote genes under positive selection, whilst non-bold letters denote genes under neutral or purifying selection. Light gray shading indicates genes under positive selection in IRRB and under neutral or purifying selection in IRSB. Dark gray shading indicates genes that are under positive selection in IRRB for which orthologs are absent in all IRSB (see Methods). Click here for file

Additional file 2 **Alignment of sets of orthologs in ionizing-radiation-resistant bacteria (IRRB)**. Sets of orthologs present in all IRRB were aligned with CLUSTAL W following detection with MultiParanoid (see Methods). Click here for file

Additional file 3 **Alignment of sets of orthologs in ionizing-radiation-resistant bacteria (IRRB)**. Sets of orthologs present in all IRRB were aligned with CLUSTAL W following detection with MultiParanoid (see Methods). Click here for file

Additional file 4 **Alignment of sets of orthologs in ionizing-radiation-resistant bacteria (IRRB)**. Sets of orthologs present in all IRRB were aligned with CLUSTAL W following detection with MultiParanoid (see Methods). Click here for file

Additional file 5 **Alignment of sets of orthologs in ionizing-radiation-resistant bacteria (IRRB)**. Sets of orthologs present in all IRRB were aligned with CLUSTAL W following detection with MultiParanoid (see Methods). Click here for file

Additional file 6 **Alignment of sets of orthologs in ionizing-radiation-resistant bacteria (IRRB)**. Sets of orthologs present in all IRRB were aligned with CLUSTAL W following detection with MultiParanoid (see Methods). Click here for file

Additional file 7 **Alignment of sets of orthologs in ionizing-radiation-resistant bacteria (IRRB)**. Sets of orthologs present in all IRRB were aligned with CLUSTAL W following detection with MultiParanoid (see Methods). Click here for file

Additional file 8 **Alignment of sets of orthologs in ionizing-radiation-resistant bacteria (IRRB)**. Sets of orthologs present in all IRRB were aligned with CLUSTAL W following detection with MultiParanoid (see Methods). Click here for file

Additional file 9 **Alignment of sets of orthologs in ionizing-radiation-sensitive bacteria (IRSB) for which there are orthologs in all IRRB**. Sets of orthologs present in all IRSB, and for which there are orthologs in all IRRB, were aligned with CLUSTAL W following detection with MultiParanoid (see Methods). Click here for file

Additional file 10 **Alignment of sets of orthologs in ionizing-radiation-sensitive bacteria (IRSB) for which there are orthologs in all IRRB**. Sets of orthologs present in all IRSB, and for which there are orthologs in all IRRB, were aligned with CLUSTAL W following detection with MultiParanoid (see Methods). Click here for file

Additional file 11 **Alignment of sets of orthologs in ionizing-radiation-sensitive bacteria (IRSB) for which there are orthologs in all IRRB**. Sets of orthologs present in all IRSB, and for which there are orthologs in all IRRB, were aligned with CLUSTAL W following detection with MultiParanoid (see Methods). Click here for file
